# Pharmacological activities of esculin and esculetin: A review

**DOI:** 10.1097/MD.0000000000035306

**Published:** 2023-10-06

**Authors:** Ting Cai, Bin Cai

**Affiliations:** a Department of Nephrology, The Affiliated Wuxi People’s Hospital of Nanjing Medical University, Wuxi People’s Hospital, Wuxi Medical Center, Wuxi, China; b Department of Anorectal Surgery, Wuxi Hospital Affiliated to Nanjing University of Chinese Medicine, Wuxi, China.

**Keywords:** *Cortex Fraxini*, coumarin, esculetin, esculin

## Abstract

Esculin and esculetin are 2 widely studied coumarin components of *Cortex Fraxini*, which is a well-known herbal medicine with a 2000-year history. In vivo and in vitro studies have demonstrated that both have a variety of pharmacological activities, including antioxidant, anti-tumor, anti-inflammatory, antibacterial, antidiabetic, immunomodulatory, anti-atherosclerotic, and so on. Their underlying mechanisms of action and biological activities include scavenging free radicals, modulating the nuclear factor erythroid 2-related factor 2 pathway, regulating the cell cycle, inhibiting tumor cell proliferation and migration, promoting mitochondrial pathway apoptosis, inhibiting the NF-κB and MAPK signaling pathways, regulating CD4^+^ T cells differentiation and associated cytokine release, inhibiting vascular smooth muscle cells, etc. This review aims to provide comprehensive information on pharmacological studies of esculin and esculetin, which is of noteworthy importance in exploring the therapeutic potential of both coumarin compounds.

## 1. Introduction

*Cortex Fraxini* (also known as Qinpi in Chinese) is a well-known herbal medicine that belongs to the “heat-clearing” category in traditional Chinese medicine. It is the dried bark of *Fraxinus rhynchophylla* Hance, *Fraxinus chinensis* Roxb., *Fraxinus szaboana* Lingelsh., and *Fraxinus stylosa* Lingelsh. As early as the Donghan Dynasty in China, about 2000 years ago, it was used to treat diarrhea and bacillary dysentery. In addition, *Cortex Fraxini* has been used separately to treat conjunctivitis for as long as 1000 years up to the present day. *Cortex Fraxini* is also an important traditional Chinese herbal medicine used for the treatment of gout and hyperuricemia.^[1]^

Modern pharmacological studies have shown that coumarin components are the active constituents of *Cortex Fraxini*.^[2]^ Coumarins (1-benzopyran-2-one) are natural compounds of the benzopyrone class that are widely distributed in plants, as well as many species of fungi and bacteria.^[3]^ Various promising pharmacological activities of coumarins were reported by many in vivo and in vitro studies, including anti-inflammatory,^[4]^ antioxidant,^[5]^ antibacterial,^[6]^ anti-tumor,^[7]^ and so on. As natural products, coumarins are considered promising as complementary and alternative medicines.

Esculin and esculetin are the most studied coumarin components in *Cortex Fraxini*. Esculin (6-beta-glucoside-7-hydroxycoumarin, molecular formula: C15H16O9) is a glycosidic coumarin derivative. The 2 parts of the molecule (glucose and 7-hydroxycoumarin) are linked by an ester linkage through oxygen. Notably, the oral bioavailability of esculin is only 0.62%.^[8]^ Esculin can be enzymatically hydrolyzed at the 8-glucose linkage to yield 2 products, esculetin and glucose. Esculetin (6,7-dihydroxy-2-chromenone, molecular formula: C9H6O4) is one of the simplest coumarins with 2 hydroxyl groups at carbons 6 and 7, and is the aglycone metabolite of esculin.^[9]^ The average oral bioavailability of esculetin is 19%, which is significantly higher than that of esculin.^[10]^

A literature search was conducted using PubMed, Web of Science, and CNKI databases through various combinations of keywords. The primary keywords for the literature search included “esculin,” “esculetin,” “*Cortex Fraxini*,” “pharmacology,” “tumor,” “oxidative stress,” “inflammation,” “bacteria,” and “diabetes.” Comprehensive information is intended to be provided by this review regarding the research progress of esculin and esculetin in pharmacology, which is of remarkable significance in association with the exploration of the therapeutic potential of both coumarin compounds.

## 2. Pharmacological activities of esculin

### 2.1. Anti-inflammatory activity of esculin

**Figure 1.**
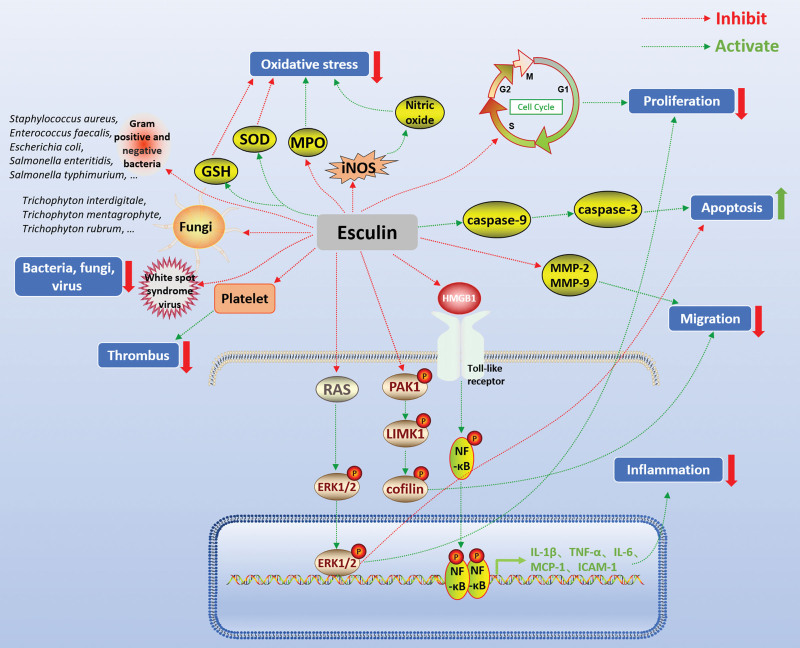
Pharmacological activities of esculin. Green arrows indicate activating effects and red flat heads indicate inhibiting effects. ERK = extracellular regulatory protein kinase, GSH = glutathione, ICAM-1 = intercellular cell adhesion molecule-1, IL-1β = interleukin-1β, iNOS = inducible nitric oxide synthase, LIMK1 = LIM domain kinase 1, MCP-1 = monocyte chemoattractant protein-1, MMP-2 = matrix metalloproteinase-2, MPO = myeloperoxidase, NF-κB = nuclear factor-kappa B, PAK1 = protein-activated kinase 1, RAS = rat sarcoma virus, SOD = superoxide dismutase, TNF-α = tumor necrosis factor-α.

Studies have shown that esculin inhibits the inflammatory response in ulcerative colitis,^[11]^ arthritis,^[12]^ acute lung injury,^[13]^ acute kidney injury,^[14]^ and other diseases. Esculin was found to significantly alleviate the symptoms of colitis and suppress the expression of inflammatory factors including inducible nitric oxide synthase (iNOS), tumor necrosis factor-α (TNF-α), and interleukin-1β (IL-1β) in vivo and in vitro. Notably, esculin attenuated the activity of nuclear factor-kappa B (NF-κB) signaling pathway in gut tissue with colitis and lipopolysaccharide (LPS)-stimulated RAW264.7 macrophages. These results demonstrated that the protection of esculin against colitis may be due to its function in the regulation of the NF-κB pathway.^[11]^ Zheng et al^[12]^ demonstrated that esculin attenuated the inflammatory response of adjuvant-induced arthritis in rats by inhibiting several pro-inflammatory cytokines such as IL-1β and TNF-α. In LPS-challenged mice, esculin was shown to significantly attenuate LPS-induced lung pathological injury, reduce pro-inflammatory cytokine levels in the bronchoalveolar lavage fluid, and suppress the activation of the NF-κB signaling in the lung. Furthermore, esculin impaired neutrophil migration and chemotaxis in vitro by suppressing the protein-activated kinase 1/LIM domain kinase 1/cofilin signaling axis.^[13]^ Zhang et al^[15]^ also demonstrated that pretreatment with esculin (20 and 40 mg/kg) attenuated inflammation, reduced neutrophil numbers, and decreased the expression of TNF-α, IL-1β, and IL-6 in the bronchoalveolar lavage fluid of LPS-induced mice with acute lung injury. Moreover, researchers suggested that its anti-inflammatory mechanism may be through the inhibition of the Toll-like receptor/NF-κB signaling pathway. Similarly, esculin treatment significantly reduced the release of pro-inflammatory factors in LPS-induced acute kidney injury in mice, including IL-1β, IL-6, TNF-α, monocyte chemoattractant protein-1, and intercellular cell adhesion molecule-1.^[14]^ In addition, esculin was shown to protect against LPS-induced acute kidney injury by inhibiting the high mobility group box 1/Toll-like receptor 4 inflammatory pathway. The expressions of the inflammatory pathway proteins P2X7, high mobility group box 1, Toll-like receptor 4, and myeloid differentiation primary response 88 at both the mRNA and protein levels were downregulated by esculin treatment in the kidney tissue of LPS-challenged mice. The above results suggested that esculin mainly inhibits the NF-κB signaling pathway, thereby reducing the expression of pro-inflammatory cytokines such as IL-1β, IL-6, TNF-α, etc., and thus exerting anti-inflammatory effects (Fig. 1).

### 2.2. Anti-tumor activity of esculin

Esculin has been reported to have inhibitory effects on some tumors in vitro, such as lung cancer,^[16]^ breast cancer,^[17]^ and glioblastoma,^[18]^ through regulation of cell cycle, inhibition of tumor cell proliferation, induction of mitochondrial apoptosis, etc. It was observed that esculin and its oligomeric fractions dose-dependently reduced the growth of glioblastoma U87 cells, as well as their adhesion and migration.^[18]^ Mo et al^[17]^ reported that esculin (28–900 μmol/L) significantly decreased the viability and proliferation of the triple-negative breast cancer cell line MDA-MB-231 in a dose- and time-dependent manner, and the mechanism of action involved its regulation of the cell cycle-related p53-p21 signaling pathway. p53 is a primary tumor suppressor gene that functions to inhibit proliferation and eliminate abnormal cells.^[19]^ p21 is an important negative regulator of the cell cycle that is transcriptionally controlled by p53 pathways.^[20]^ In addition, esculin (0.2, 0.6, and 1.8 mmol/L) was found to inhibit the migration and invasion of nasopharyngeal carcinoma HNE-3 cells by reducing the levels of matrix metalloproteinase-2 (MMP-2) and MMP-9 proteins.^[21]^ MMPs are a family of zinc-dependent proteolytic endopeptidases with extracellular matrix remodeling and degradation properties and have long been implicated in cancer initiation, tumor growth and metastasis.^[22]^ Researchers discovered that the activities of caspase-3 and caspase-9 increased in human A549 lung cancer cells after treatment with esculin.^[16]^ As the main executioner of apoptosis, caspase-3 is cleaved and activated during the early stages of apoptosis to execute apoptosis by cleaving targeted cellular proteins.^[23]^ Caspase-9 is a key player in the mitochondrial apoptosis pathway, and activated caspase-9 cleaves downstream caspases such as caspase-3, caspase-6, and caspase-7.^[24]^ Furthermore, it was found that the rat sarcoma virus/extracellular regulatory protein kinase (ERK) signaling pathway was downregulated by esculin treatment in A549 cells,^[16]^ which plays a critical role in tumor proliferation, invasion, and metastasis.^[25]^ Since the studies on the anti-tumor effects of esculin are mainly focused on in vitro experiments, more in vivo studies are needed to further investigate its mechanism of action.

### 2.3. Antioxidant activity of esculin

There are many highly reactive molecules called free radicals in the body, including reactive oxygen species (ROS), such as superoxide anion radical, hydroxyl radical, and hydrogen peroxide, and reactive nitrogen species, such as nitric oxide, peroxynitrite, and nitroxyl anion. When produced or accumulated in excess, these highly reactive molecules can cause an oxidative imbalance, resulting in oxidative damage to tissues and cells.^[26]^ The 2,2-diphenyl-1-picrylhydrazyl assay showed that esculin has an efficient free radicals scavenging ability (EC50 = 0.141 μM).^[27]^ The antioxidant capacity of esculin was demonstrated in gastric tissue subjected to ethanol challenge, where it was able to significantly reduce the production of nitric oxide by decreasing the activity of iNOS.^[28]^ Witaicenis et al^[29]^ demonstrated that administration of esculin (25 mg/kg) significantly reduced myeloperoxidase activity in the colonic tissue of rats with colitis. As a prominent heme peroxidase, myeloperoxidase can mediate oxidative stress by promoting the production of ROS and regulating inflammation-related signaling pathways. In human neuroblastoma SH-SY5Y cells, esculin was found to reduce dopamine-induced ROS overproduction, and enhance superoxide dismutase (SOD) and glutathione (GSH) activities.^[30]^ SOD is an enzymatic antioxidant that catalyzes the dismutation of superoxide anion to molecular oxygen and hydrogen peroxide, which is then enzymatically scavenged by GSH. GSH is the major hydrophilic antioxidant that protects cells from free radicals.^[31]^ In addition, esculin has also demonstrated its antioxidant activity in studies related to acute lung injury,^[32]^ diabetic nephropathy,^[33]^ bioallethrin-induced toxicity,^[34]^ etc. In short, the antioxidant effects of esculin have been widely reported. Its mechanism of action may be due to its direct antioxidant capacity and modulation of oxidative stress-related enzyme activities.

### 2.4. Antibacterial, antifungal, and antiviral activities of esculin

Studies have shown that esculin has certain antibacterial activity. Mokdad-Bzeouich et al^[35]^ investigated the antibacterial activity of esculin and its oligomer fractions by in vitro tests and found them to be inhibitory against the Gram-positive bacteria *Staphylococcus aureus* and *Enterococcus faecalis*, the Gram-negative bacteria *Escherichia coli, Salmonella enteritidis, Salmonella typhimurium*, and several *E coli* multiresistant variants. In addition to bacteria, esculin has been shown to have antibiosis effects against a variety of fungi including *Trichophyton interdigitale, Trichophyton mentagrophyte, Trichophyton rubrum, Trichophyton soudanens, Trichophyton tonsurans, Microsporum canis, Aspergillus fumigatus*, and *Scopulariopsis brevicaulis*.^[36]^ Moreover, a recent study found that esculin could prevent and treat white spot syndrome virus infection.^[37]^ It was demonstrated that esculin (100 μM) increased the survival rate of white spot syndrome virus-infected shrimps by 59% and reduced the virus copy number in vivo over 90%. Studies have shown that esculin can be distributed in the body in a rapid and even manner.^[38]^ However, it is worth noting that the first pass effect of esculin is serious. Therefore, how to improve its bioavailability is the key to apply its antibiotic properties.

### 2.5. Antidiabetic activity of esculin

The antidiabetic effects of esculin have been confirmed in several in vivo studies. Kang et al^[39]^ reported that oral administration of esculin, 20 mg/kg/day for 2 weeks, resulted in a significant increase in food and water intake and a significant decrease in blood glucose levels and hepatic glucose-6-phosphatase expression in streptozotocin-induced diabetic mice. Moreover, treatment with esculin could reduce diabetes-induced renal damage by alleviating oxidative stress and suppressing inflammation. Yao and colleagues reached a similar conclusion that esculin lowered blood glucose and prevented diabetic kidney damage in diabetic mice.^[40]^ Additionally, esculin (40 mg/kg) was found to ameliorate insulin resistance and increase the activities of glutathione peroxidase, SOD, and catalase in the pancreas and liver of diabetic mice. Esculin was also found to improve diabetic neuropathy in experimental diabetic rats.^[41]^ Overall, the antidiabetic activity of esculin maybe essentially attributed to its antioxidant and anti-inflammatory effects.

### 2.6. Antithrombotic activity of esculin

There is evidence that esculin may have antithrombotic potential. Ahmad et al^[42]^ reported that pretreatment with esculin or esculin pentasulfate reduced thrombus weight and length in a rat model of thrombosis induced by inferior vena cava ligation. In addition, both esculin and esculin pentasulfate prolonged the activated clotting time, clotting time, and clotting rate in the experimental rats compared to the control and vehicle groups, with esculin pentasulfate exhibiting a more potent anticoagulant effect than esculin. Furthermore, esculin pentasulfate was found to have an anti-platelet effect. It was suggested that modifying the structure of esculin would help to enhance its antithrombotic activity.

## 3. Pharmacological activities of esculetin

### 3.1. Anti-tumor activity of esculetin

**Figure 2.**
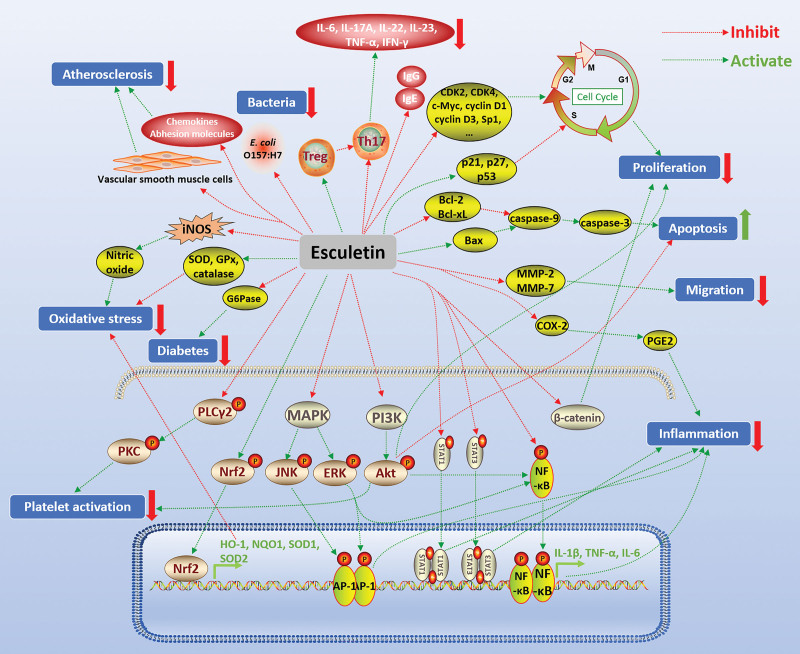
Pharmacological activities of esculetin. Green arrows indicate activating effects and red flat heads indicate inhibiting effects. Akt = protein kinase B, AP-1 = activating protein-1, Bax = Bcl-2-associated X protein, Bcl-2 = B-cell lymphoma 2, Bcl-xL = B-cell lymphoma-extra large, COX-2 = cyclooxygenase-2, ERK = extracellular regulatory protein kinase, G6Pase = glucose-6-phosphatase, GPx = glutathione peroxidase, GSH = glutathione, HO-1 = heme oxygenase-1, IgE = immunoglobulin E, IFN-γ = interferon-γ, IL-1β = interleukin-1β, iNOS = inducible nitric oxide synthase, JNK = c-Jun N-terminal kinase, MAPK = mitogen-activated protein kinase, MMP-2 = matrix metalloproteinase-2, NF-κB = nuclear factor-kappa B, NQO1 = NAD(P)H quinone oxidoreductase 1, Nrf2 = nuclear factor erythroid 2-related factor 2, PGE2 = prostaglandin E2, PI3K = phosphatidyl inosine 3 kinase, PKC = protein kinase C, PLCγ2 = phospholipase Cγ2, SOD = superoxide dismutaese, Sp1 = specificity protein 1, STAT1 = signal transducer and activator of transcription 1, Th17 = T helper type 17, TNF-α = tumor necrosis factor-α, Treg = regulatory T cell.

Many in vivo and in vitro experiments have shown that esculetin has significant anti-tumor pharmacological activities against a variety of tumors, such as colorectal cancer,^[43]^ gastric cancer,^[44]^ hepatocellular carcinoma,^[45]^ oral squamous cell carcinoma,^[46]^ leukemia,^[47]^ endometrial cancer,^[48]^ lung cancer,^[49]^ pancreatic cancer,^[50]^ prostate cancer,^[51]^ laryngeal cancer,^[52]^ malignant melanoma,^[53]^ breast cancer,^[54]^ cervical cancer,^[55]^ osteosarcoma,^[56]^ etc. The anti-tumor mechanism of esculetin mainly included inhibiting tumor cell proliferation by regulating the tumor cell cycle, inducing apoptosis through the mitochondrial pathway, and inhibiting tumor cell migration. It was found that esculetin induced the arrest of the cell cycle at the G0/G1 phase in human colorectal cancer LoVo cells. After treatment with esculetin, the protein expression of p53, p27, and p21 was increased in LoVo cells, and the protein expression of cyclin D1 and specificity protein 1 was decreased.^[43]^ Cyclin D1 plays a central role in the pathogenesis of cancer by determining uncontrolled cell proliferation and is a key regulator of the cell cycle.^[57]^ Specificity protein 1 has also been shown to play an important role in cell growth, differentiation, apoptosis, and carcinogenesis.^[58]^ In human leukemia HL-60 cells, esculetin dose-dependently suppressed the proliferation and induced G0/G1 cell cycle arrest, which was accompanied by the suppression of cyclin D1, cyclin D3, cyclin-dependent kinases 2, and CDK4.^[47]^ Furthermore, esculetin was shown to block the cell cycle at the S-phase in hepatocellular carcinoma SMMC-7721 cells,^[45]^ and to decrease the mRNA and protein levels of c-Myc and cyclin D1,^[59]^ which are closely associated with the irregular proliferation of tumor cells. It was suggested that inhibition of the Wnt/β-catenin pathway by esculetin played an important role in its suppressive effect on proliferation (Fig. 2).^[59]^

The mitochondrial pathway is the major pathway of physiological cell death in vertebrate animals. Mitochondrial intermembrane space proteins, especially cytochrome c, are released into the cytosol via mitochondrial outer membrane permeabilization. Upon release of cytochrome c from the mitochondria, apoptotic protease activator-1 activates caspase-9 in response to various stimuli. Activated caspase-9 cleaves effector caspases, mainly caspase-3, leading to cell death.^[60]^ Mitochondrial outer membrane permeabilization can be regulated by proteins of the B-cell lymphoma 2 (Bcl-2) family, including anti-apoptotic proteins, such as Bcl-2 and B-cell lymphoma-extra large, and pro-apoptotic proteins, such as Bcl-2 antagonist/killer and Bcl-2-associated X protein (Bax). The pro- and anti-apoptotic Bcl-2 proteins function through protein–protein interactions in soluble and membrane-associated states.^[61,62]^ In LoVo cells, esculetin treatment (200, 400, and 600 μM) activated caspase-3, caspase-7, and caspase-9, upregulated the expression of Bax, and downregulated the expression of Bcl-2 in a dose-dependent manner.^[43]^ Wang et al^[44]^ found that esculetin (850 μM) induced significant apoptosis of gastric cancer cell lines (MGC-803, HGC-27, and BGC-823) by triggering the activation of the mitochondrial apoptotic pathway. Esculetin reduced mitochondrial outer membrane permeabilization, increased Bax/Bcl-2 ratio, activated caspase-3 and caspase-9, and increased mitochondrial cytochrome c release in MGC-803 cells. Furthermore, it was suggested that esculetin promoted apoptosis by downregulating the insulin-like growth factor-1/phosphatidyl inosine 3 kinase/protein kinase B (Akt) pathway. In oral squamous cell carcinoma cell lines (HN22 and HSC2), esculetin also induced apoptosis by upregulating Bax expression, downregulating B-cell lymphoma-extra large expression, and cleaving caspase-3.^[46]^ In addition, esculetin was found to effectively inhibit the phosphatidyl inosine 3 kinase/Akt signaling pathway in HN22 and HSC2 cells, which plays an important role in numerous cellular processes, including inhibition of apoptosis, stimulation of cell growth, and modulation of cellular metabolism.^[63]^

Esculetin was demonstrated to inhibit the migration of many tumor cells, including human laryngeal cancer Hep-2 cells,^[52]^ cervical cancer HeLa cells,^[55]^ prostate cancer PC3 cells,^[51]^ etc. In human colorectal cancer HCT-116 cells, esculetin (20 µg/mL) alone significantly inhibited cell migration and had a synergistic effect when used in combination with 5-fluorouracil (10 µg/mL).^[64]^ It was further found that tumor migration-related target molecules, MMP-2 and MMP-7, were significantly downregulated by esculetin treatment in HCT-116 cells. The expression of MMP-2 was also inhibited by esculetin (10–100μM) in osteosarcoma LM8 cells.^[56]^ It is generally accepted that cellular invasion is attributed to the synthesis and secretion of MMPs with protein hydrolytic activity by tumor cells to degrade the extracellular matrix and promote metastasis.^[65]^

### 3.2. Antioxidant activity of esculetin

The molecular structure of esculetin has 2 hydroxyl groups, which is believed to be the structural basis of its antioxidant properties. It has been proven that the activity of coumarins is correlated with the number of hydroxyl groups, and the number of hydroxyl groups is, in turn, directly correlated with the effects of ROS suppression.^[66]^ Kim et al^[67]^ reported that in Chinese hamster lung fibroblast V79-4 cells after hydrogen peroxide treatment, the intracellular ROS scavenging activity of esculetin was 40% at 1 µg/mL and 75% at 10 µg/mL. The inhibitory effect of esculetin on nitric oxide production and iNOS expression was also observed in lipoteichoic acid-induced macrophage cells.^[68]^ It was found that esculetin preconditioning (1.25–5 µM) significantly attenuated hydrogen peroxide-induced DNA damage and apoptosis in C2C12 myoblast cell line by suppressing intracellular ROS accumulation. In addition, treatment with esculetin effectively increased the phosphorylation of nuclear factor erythroid 2-related factor 2 (Nrf2) and the expression of NAD(P)H quinone oxidoreductase 1 (NQO1) in C2C12 cells.^[69]^ Accumulating evidence suggests that Nrf2 may be a critical regulator of the cellular antioxidant response for protection against oxidative stress-induced DNA damage and apoptosis. Nrf2 is an essential transcription factor that regulates multiple antioxidant defense genes through binding to antioxidant responsive elements.^[70]^ NQO1 is an antioxidant enzyme that catalyzes the 2-electron reduction of several different classes of quinone-like compounds, including quinones, quinone imines, nitro aromatics, and azo dyes.^[71]^ Zhang et al^[72]^ also reported that esculetin played an antioxidant role by strongly induced Nrf-2 translocation to the nucleus, which in turn increased the mRNA expression levels of antioxidant genes heme oxygenase-1, NQO1, SOD1, and SOD2 in hydrogen peroxide-treated human corneal epithelial cells. HO-1 can catalyze the degradation of heme to form carbon monoxide, ferrous iron and biliverdin. Biliverdin is then converted to bilirubin by biliverdin reductase, and both biliverdin and bilirubin act as antioxidants by scavenging or neutralizing ROS.^[73]^ In vivo experiments also confirmed the antioxidant effects of esculetin. In a mouse model of acute lung injury, treatment with esculetin (40 mg/kg) not only reduced the hepatic levels of nitric oxide, 3,4-methylenedioxyamphetamine, and ROS, but also improved the activity of SOD and the expression of Nrf2.^[74]^ In addition, esculetin (100 and 500 mg/kg) significantly increased the activities of the antioxidant enzymes glutathione peroxidase, SOD, and catalase in the liver of carbon tetrachloride-induced rats.^[75]^ Since oxidative stress is a known trigger for the onset of many diseases, the free radicals scavenging ability of esculetin is also associated with other diseases such as inflammation, cancer, etc.

### 3.3. Anti-inflammatory activity of esculetin

The anti-inflammatory effects of esculetin have been widely reported in vitro and in vivo. Hong et al^[76]^ reported that esculetin (2, 6, and 12 μg/mL) concentration-dependently inhibited the production of nitric oxide and prostaglandin E2, as well as the expression of iNOS and cyclooxygenase-2 (COX-2) in LPS-activated RAW 264.7 macrophages. In addition, esculetin significantly suppressed the production of inflammatory cytokines such as TNF-α and IL-1β, by attenuating the activation of the NF-κB pathway in RAW 264.7 macrophages. Cheng et al^[77]^ proposed that esculetin protects against early sepsis by attenuating inflammation through inhibition of NF-κB and STAT1/STAT3 signaling pathway. It was observed that esculetin (10–40 μmol/L) inhibited the production of pro-inflammatory factors such as IL-1β, IL-6, TNF-α, iNOS, and C-C motif chemokine ligand 2 in LPS-stimulated macrophages. Suppressed expression of the above-mentioned cytokines by esculetin treatment was also found in the lungs of septic mice. In addition, a recent study indicated that esculetin was also able to inhibit the activation of NLR family pyrin domain containing 3 inflammasome in LPS-induced RAW264.7 cells.^[78]^ Other in vivo experiments have similarly confirmed the anti-inflammatory activity of esculetin. In LPS-induced mice with acute lung injury, pretreatment with esculetin (20 and 40 mg/kg) significantly attenuated LPS-induced histopathologic changes, decreased inflammatory cell infiltration, and reduced the production of pro-inflammatory cytokines (TNF-α, IL-1β, and IL-6) in lung tissue. Furthermore, esculetin suppressed the phosphorylation of Akt, ERK, and NF-κB, and inhibited the expression of RAR-related orphan receptor-γ and IL-17 in the lung tissue of mice with acute lung injury. Esculetin has also been shown to have anti-inflammatory effects on colitis. It was reported that esculetin promoted a reduction in lesion extent accompanied by reduced diarrhea incidence and restored glutathione levels in the colitis model.^[79]^ In addition, the expression of pro-inflammatory mediators (COX-2, iNOS and cytokine-induced neutrophil chemoattractant-3) in the inflamed colon of rats was reduced by rectal administration of esculetin (100 and 200 μM).^[80]^ In dextran sulfate sodium-induced colitis mice, treatment with esculetin (20 mg/kg) inhibited the release of TNF-α and IL-6, blocked the NF-κB signaling pathway, and suppressed the phosphorylation of mitogen-activated protein kinases (MAPKs) such as p38, c-Jun N-terminal kinase, and ERK, which in turn inhibited the downstream activation of activating protein-1.^[78]^ Suppression of the activating protein-1 pathway reduced the expression of inflammatory cytokines and chemokines.^[81]^ As studies continue, we believe esculetin has the potential to be a promising anti-inflammatory agent.

### 3.4. Immunomodulatory activity of esculetin

Studies have shown that esculetin may act on both humoral and cellular mediated immunity. Jeong et al^[82]^ found that oral administration of esculetin (2, 10, and 50 mg/kg) decreased serum immunoglobulin E, IgG2a, and histamine levels, and suppressed the production of T helper type 17 (Th17)-, Th2-, and Th1-related cytokines such as TNF-α, interferon-γ, IL-4, IL-13, IL-31, and IL-17 in atopic skin rats. In an ovalbumin-induced asthmatic mouse model, treatment with esculetin (20 and 40 mg/kg) dramatically reduced the level of immunoglobulin E in the serum and decreased the production of Th2- and Th17-related cytokines, including IL-4, IL-5, IL-13, and IL-17A. Furthermore, esculetin inhibited the percentage of Th17 cells in the spleen and reduced protein levels of GATA-binding protein 3 and RAR-related orphan receptor-γ in lung tissue.^[83]^ Chen et al^[84]^ reported that esculetin (50 and 100 mg/kg) reduced the frequency of CD8^+^ CD44^high^ CD62^low^ effector T cells in psoriatic mice. In contrast, it promoted the differentiation of CD4^+^ CD25^-^ T cells into CD4^+^ Foxp3^+^ regulatory T cells (Tregs) in vitro and increased the frequency of CD4^+^ Foxp3^+^ Tregs in both lymph nodes and spleen of psoriatic mice. Additionally, the mRNA levels of pro-inflammatory cytokines in psoriatic mouse, including IL-6, IL-17A, IL-22, IL-23, TNF-α, and interferon-γ, were dramatically decreased by esculetin treatment. Due to the immunomodulatory activity of esculetin, its potential therapeutic role in the treatment of immune disorders is highly desirable.

### 3.5. Anti-atherosclerotic and anticoagulant activities of esculetin

Researchers proposed esculetin as a promising functional molecule that may have preventive or therapeutic functions in the treatment of atherosclerosis. Wang et al^[85]^ suggested that esculetin played an anti-atherosclerotic role by reducing the blood triglyceride level and preventing the proliferation of vascular smooth muscle cells and the production of MMP-9. Esculetin treatment also inhibited the oxidation of low-density lipoprotein as well as the secretion of adhesion factors and chemokines, and increased the efflux level of high-density lipoprotein cholesterol. The proliferation and infiltration of vascular smooth muscle cells from the media into the intima is an essential step in the pathophysiology of atherosclerosis and restenosis. It was demonstrated that esculetin (1–100µM) inhibited the DNA synthesis and cell number of vascular smooth muscle cells in a concentration-dependent manner.^[86]^ Yun et al^[87]^ also reported that treatment of vascular smooth muscle cells with esculetin (25–300 µg/mL) resulted in a significant inhibition of cell growth and DNA synthesis in a concentration-dependent manner. Furthermore, esculetin induced a G1 phase cell cycle arrest in vascular smooth muscle cells, which was due to inhibition of cyclin D1/CDK4 and cyclin E/cyclin-dependent kinases 2 complexes through activation of the p38 MAPK pathway. In addition, it was demonstrated that esculetin may be an important therapeutic agent for preventing thromboembolic diseases.^[88]^ Esculetin treatment (10–80 μM) exhibited strong effectiveness for inhibiting platelet aggregation stimulated by arachidonic acid and collagen. Furthermore, esculetin decreased the adenosine triphosphate release and P-selectin expression and inhibited the phosphorylation of phospholipase Cγ2, protein kinase C, and Akt in collagen-activated human platelets. Phospholipase Cγ2/protein kinase C/Akt activation is involved in collagen-dependent signaling in platelets.^[89]^ In experimental mice, esculetin substantially prolonged the closure time of whole blood and significantly increased the occlusion time in thrombotic platelet plug formation.

### 3.6. Antidiabetic activity of esculetin

Esculetin is believed to have the potential to treat diabetes and its complications by promoting glucose degradation, inhibiting glucose production, increasing insulin levels, and improving insulin sensitivity. Choi et al^[90]^ reported that diabetic mice fed a diet containing 100 mg/kg esculetin for several weeks tended to have lower blood glucose levels and showed a significant decrease in glycosylated hemoglobin. In addition, treatment with esculetin led to a reduction of glucose-6-phosphatase (G6Pase) activity in diabetic mice, as well as a significant decrease in the G6Pase/glucokinase ratio compared to control diabetic mice. The decrease of hepatic G6Pase activity and G6Pase/glucokinase ratio contributed to the inhibition of hepatic glucose production. In addition, esculetin was found to prevent nonalcoholic fatty liver disease in diabetic mice by reducing the expression of genes involved in lipid synthesis and inflammation. Prabakaran et al^[91]^ found that oral administration of esculetin (10–40 mg/kg) significantly reduced blood glucose levels and increased plasma insulin levels in diabetic rats, and ameliorated diabetes-induced tissue damage in the liver and kidney by attenuating hyperglycemia-mediated oxidative stress. Furthermore, esculetin has been demonstrated to have protective effects against diabetes-induced complications such as nerve damage,^[92]^ and vascular dysfunction.^[93]^

### 3.7. Antibacterial activity of esculetin

Many natural coumarins have been demonstrated to have antibacterial activity including esculetin.^[94]^ *E coli* O157:H7 is a bacterium that normally lives in the human gut, but can cause severe intestinal infection.^[95]^ Duncan et al^[96]^ reported that esculetin significantly inhibited the survival of *E coli* O157:H7 during in vitro incubation with human feces. Moreover, it was found that esculetin repressed Shiga-like toxin stx2 gene in *E coli* O157:H7 and dose-dependently prolonged C. elegans survival in the presence of *E coli* O157:H7, indicating that esculetin could attenuate the virulence of *E coli* O157:H7 in vivo.^[97]^

## 4. Conclusion

As 2 widely studied coumarin components, in vivo and in vitro researches have demonstrated that esculin and esculetin have a variety of pharmacological activities, including antioxidant, anti-inflammatory, anti-tumor, immunomodulatory, antidiabetic, antibacterial, antifungal, antiviral, anti-atherosclerotic, anticoagulant, and so on. Among them, their prominent antioxidant effects play an important role in many kinds of diseases, and scavenging free radicals and regulating the Nrf2 pathway may be the key to their antioxidant properties. In addition, in vitro studies have found that they can exert anti-tumor effects by regulating the cell cycle, inhibiting cell proliferation and migration, and promoting mitochondrial pathway apoptosis. However, more in vivo experiments are needed to further explore their anti-tumor effects and mechanisms. Their anti-inflammatory effects have also been widely reported, where inhibition of the NF-κB pathway may be the basis of their anti-inflammatory properties, and other pathways such as the STAT1/STAT3, MAPK, and COX-2/PEG2 pathway are also worthy of attention. Furthermore, their immunomodulatory role in the differentiation of CD4^+^ T cells, such as Th17 and Treg, and the release of associated cytokines contribute to their anti-inflammatory effects. In addition, the antibiosis effects of esculetin against bacteria and fungi as well as the inhibitory effect of esculetin on vascular smooth muscle cells are also of interest. In conclusion, as natural products, esculin and esculetin are worthy of future investigation, and both are promising as potential therapeutic agents for clinical applications.

## Author contributions

**Conceptualization:** Bin Cai.

**Formal analysis:** Ting Cai, Bin Cai.

**Funding acquisition:** Ting Cai, Bin Cai.

**Supervision:** Bin Cai.

**Writing – original draft:** Ting Cai, Bin Cai.

**Writing – review & editing:** Bin Cai.
